# Developmental Changes in Phenolic Composition and Condensed Tannin Structure in the Skins of Three Table Grape Cultivars: ‘Rosetta’, ‘Shooting Star’, and ‘Hongju Seedless’

**DOI:** 10.3390/foods15193482

**Published:** 2026-09-29

**Authors:** Byeong Hyeon Yun, Dongjun Im, Su In Mo, Suh-Hyun Ryu, Seon-Hwa Bae, Jung-Ho Noh, Youn-Young Hur, Jeong Sil Choi, Hyungmin Rho, In-Girl Baek

**Affiliations:** 1 Fruit Research Division, National Institute of Horticultural & Herbal Science, Wanju 55365, Republic of Korea; ybh92@korea.kr (B.H.Y.); bae209@korea.kr (S.-H.B.);; 2 Department of Agriculture, Forestry, and Bioresources and Research Institute of Agriculture and Life Sciences, Seoul National University, Seoul 08826, Republic of Korea; 3 Plant Science and Biotechnology Institute and Plant Genomics and Breeding Institute, Seoul National University, Seoul 08826, Republic of Korea

**Keywords:** table grape, proanthocyanidin, flavan-3-ol, thioacidolysis, mean degree of polymerization

## Abstract

This study investigated developmental changes in phenolic compounds and condensed tannin structure in the berry skins of three table grape cultivars, ‘Rosetta’ (RT), ‘Shooting Star’ (SS), and ‘Hongju Seedless’ (HS), from flowering to harvest. Spectrophotometric analyses were used to quantify total phenolic, proanthocyanidin, polymeric tannin, and anthocyanin contents, while high-performance liquid chromatography coupled with thioacidolysis was applied to characterize flavan-3-ol composition and determine the mean degree of polymerization (mDP) of condensed tannins. Phenolic accumulation exhibited distinct cultivar-dependent developmental patterns. Total phenolics and flavan-3-ol monomers generally decreased as berries matured, whereas anthocyanins accumulated after veraison in RT and HS. Polymeric tannins increased during the early developmental stage and subsequently declined, while mDP showed an overall tendency to increase toward maturity, particularly in RT, although the effects of cultivar, developmental time, and their interaction on mDP were not statistically significant. HS maintained high flavan-3-ol concentrations during early development, whereas SS exhibited comparatively low phenolic accumulation together with relatively limited variation in mDP. These findings provide the first comprehensive characterization of developmental changes in flavan-3-ol composition, polymeric tannin structure, and mDP in newly developed Korean table grape cultivars and a structural basis for understanding cultivar-dependent phenolic changes during berry development.

## 1. Introduction

The rapid expansion of seedless table grape cultivars has been driven by growing consumer demand for berries that combine desirable quality attributes with the convenience of seedlessness [1,2]. In Europe and North America, seedless grapes now dominate the fresh fruit market, and breeding programs increasingly emphasize berry texture, flavor, color stability, firmness, and overall fruit quality [2,3]. Because table grapes are consumed together with the berry skin, the biochemical composition of the skin represents an important component of fruit quality. In particular, phenolic compounds and condensed tannins in the skin undergo substantial compositional and structural changes during berry development, making their characterization important for understanding cultivar-dependent differences in table grape phenolic metabolism.

Phenolic compounds constitute a major class of secondary metabolites in grape berries and contribute to color, antioxidant capacity, flavor, bitterness, and astringency [3,4,5]. Among them, condensed tannins (proanthocyanidins) are flavonoid oligomers and polymers composed primarily of (+)-catechin (C) and (−)-epicatechin (EC) subunits linked through interflavan C4–C6 or C4–C8 bonds [5,6,7,8]. Their physicochemical properties are determined not only by concentration but also by structural characteristics such as mean degree of polymerization (mDP), galloylation, and the relative abundance of terminal and extension subunits [7,9,10]. In grape berries, the skin and seeds contain most condensed tannins, which have been associated with defense against biotic and abiotic stresses, antioxidant protection, and tissue-related functions [11,12]. Therefore, characterization of condensed tannins in table grape skins requires consideration of both their abundance and structural properties rather than reliance solely on total phenolic measurements.

During berry development, flavonoid metabolism undergoes substantial temporal changes that influence the biosynthesis, accumulation, and transformation of phenolic compounds [13,14,15,16,17,18,19]. Grape berry development after flowering progresses through an early green growth phase followed by veraison, which marks the onset of ripening and is characterized by berry softening and skin color development. In the cultivars examined in this study, veraison occurred at approximately 6 WAF in ‘Rosetta’ and ‘Shooting Star’ and at approximately 7–8 WAF in ‘Hongju Seedless’, providing a developmental reference for interpreting changes in phenolic and tannin characteristics during berry development.

Condensed tannin biosynthesis generally shows the highest activity during the early stages after flowering, whereas anthocyanin accumulation begins around veraison and continues throughout ripening [13,14,15,16,17,18,19,20]. Because tannins and anthocyanins share common intermediates within the phenylpropanoid–flavonoid pathway, the transition from pre-veraison growth to post-veraison ripening may involve changes in flavonoid metabolic allocation [13,20,21], with flavonoid metabolism increasingly associated with anthocyanin biosynthesis after veraison [22,23]. Such developmental changes may influence not only tannin concentration but also polymer structure, including polymerization degree, subunit composition, and the relative abundance of terminal and extension units [12,13]. Previous studies on wine grape cultivars have reported progressive changes in tannin polymerization and flavan-3-ol composition during berry maturation, often accompanied by increasing mDP values and reductions in extractable monomeric flavan-3-ols [13,20,24]. Similar developmental changes may occur in table grapes, but their patterns and cultivar dependence remain less well characterized.

Most previous investigations of grape condensed tannins have focused on wine cultivars, particularly tannin extraction, polymerization, oxidation, and transformation during berry development and winemaking [12,13,25]. In contrast, studies on table grapes have largely focused on total phenolic content, antioxidant activity, or anthocyanin accumulation, while providing limited information regarding developmental changes in flavan-3-ol subunit composition, terminal and extension unit distribution, and mDP [3]. Consequently, the developmental dynamics of condensed tannin structure in table grape skins remain insufficiently characterized, particularly in newly bred seedless cultivars. Detailed characterization of these changes could provide a better understanding of cultivar-specific phenolic metabolism and the structural diversity of condensed tannins during berry development.

In the present study, we investigated three recently developed table grape cultivars, ‘Rosetta’, ‘Shooting Star’, and ‘Hongju Seedless’. These cultivars originate from different genetic backgrounds and exhibit distinct berry skin characteristics and phenolic profiles, making them suitable materials for comparing cultivar-dependent developmental changes in skin condensed tannins. ‘Rosetta’ originated from a cross between ‘Hongaram’ and ‘Balad’, whereas ‘Shooting Star’ originated from ‘Tano Red’ × ‘Ruby Seedless’ [26]. ‘Hongju Seedless’ originated from a cross between ‘Italia’ and ‘Perlon’ [27]. Characterization of these cultivars therefore provides an opportunity to examine the diversity of developmental tannin profiles among recently developed table grape germplasm. To the best of our knowledge, this is the first study describing developmental changes in flavan-3-ol composition, tannin polymerization, and mDP in these newly developed Korean table grape cultivars.

We hypothesized that condensed tannins in table grape skins exhibit cultivar-dependent developmental patterns in flavan-3-ol subunit composition and mDP from flowering through ripening. To test this hypothesis, we applied thioacidolysis-coupled high-performance liquid chromatography (HPLC) to berry skins collected from flowering through harvest. Thioacidolysis coupled with HPLC enables characterization of terminal and extension subunits of condensed tannins and estimation of their mean degree of polymerization (mDP), thereby providing structural information complementary to conventional phenolic measurements. The present study therefore provides fundamental information on the developmental dynamics and cultivar-dependent structural characteristics of condensed tannins in table grape skins and contributes to a better understanding of phenolic metabolism during berry development.

Because grape skin phenolics and condensed tannins are associated with sensory attributes such as astringency and bitterness, their compositional and structural characterization is also relevant to understanding grape quality. In this context, the present study contributes analytical information on phenolic compounds related to the sensory and quality characteristics of table grapes.

## 2. Materials and Methods

### 2.1. Plant Materials and Sampling Design

Grape samples of three cultivars, ‘Rosetta’ (RT), ‘Shooting Star’ (SS), and ‘Hongju Seedless’ (HS), were collected from a commercial vineyard located in Hwadongmyeon, Sangju, Gyeongsangbuk-do, Republic of Korea (36°24′ N, 128°00′ E; 280 m above sea level) in 2025. Berries were sampled at 1–2-week intervals from 1 day after flowering to harvest, covering the entire developmental period.

Veraison was observed at approximately 6 weeks after flowering (WAF) in RT and SS and at approximately 7–8 WAF in HS. Field assessment was based on the combined observation of berry softening and skin color development, with approximately 40–50% of the berries within a cluster exhibiting these ripening-associated changes. Harvest maturity was determined based on measurements of total soluble solids (TSS) and titratable acidity (TA), together with sensory assessment of sweetness and acidity.

At each sampling point, three independent biological replicates were prepared for each cultivar. Each biological replicate consisted of berries pooled from five separate clusters; therefore, a total of 15 clusters were sampled per cultivar at each sampling point. The pooled berry samples were immediately transported to the laboratory, and the skins were manually separated, frozen in liquid nitrogen, and stored at −80 °C until analysis. All spectrophotometric and HPLC measurements were performed using the three independent biological replicates (*n* = 3). The same samples were used for both spectrophotometric quantification of phenolic compounds and structural analysis of condensed tannins by HPLC.

### 2.2. Spectrophotometric Determination of Phenolic Compounds

All reagents and solvents were of analytical or HPLC grade and purchased from Sigma-Aldrich unless otherwise specified. Ultrapure water was obtained using a Milli-Q purification system. Frozen grape skins were ground under liquid nitrogen, and approximately 0.5 g of sample was extracted twice with 10 mL of acetone/water/methanol (48:36:16, *v*/*v*/*v*). The combined extracts were used for all spectrophotometric analyses.

Total phenolic content (TPC) was determined using a modified Folin–Ciocalteu method, with absorbance measured at 760 nm using a microplate reader (Multiskan GO, Thermo Fisher Scientific, Waltham, MA, USA), and results were expressed as gallic acid equivalents (GAE). Polymeric tannin content (PTC) was quantified using the bovine serum albumin (BSA) precipitation assay followed by reaction with FeCl_3_, and absorbance was recorded at 510 nm; results are expressed as tannic acid equivalents (TAE). Proanthocyanidin content (PAC) was determined using the vanillin-HCl assay with absorbance measured at 500 nm and expressed as catechin equivalents (CE). Total anthocyanin content (TAC) was measured using the pH differential method, based on absorbance differences between pH 1.0 and pH 4.5 buffers at 520 nm, and results were expressed as cyanidin-3-O-glucoside equivalents (C3GE).

### 2.3. HPLC Analysis of Flavan-3-Ols and Polymeric Tannins

#### 2.3.1. Standards, Extraction, and Fractionation

Flavan-3-ol monomer standards, including catechin (C), epicatechin (EC), gallocatechin (GC), epigallocatechin (EGC), epigallocatechin gallate (EGCg), epicatechin gallate (ECg), and catechin gallate (Cg), were purchased from Sigma-Aldrich (St. Louis, MO, USA). Procyanidin B_1_ and B_2_, used as dimeric tannin standards, were obtained from PhytoLab (Vestenbergsgreuth, Germany). Grape skin extracts were prepared using acetone/water (70:30, *v*/*v*), concentrated using an EYELA rotary evaporator, and purified using Toyopearl HW-40F (Tosoh Bioscience, Tokyo, Japan) resin column chromatography. Monomeric fractions were eluted first, followed by recovery of polymeric tannins using acetone/water (60:40, *v*/*v*).

#### 2.3.2. HPLC Analysis of Flavan-3-Ol Monomers and Dimers

Flavan-3-ol monomers and dimers were analyzed using an Agilent 1260 Infinity II system (Agilent Technologies, Santa Clara, CA, USA) equipped with a dual pump (G7111A), vial sampler (G7129A), and diode array detector (G7115A). Chromatographic separation was performed on a ZORBAX SB-C18 column (4.6 × 250 mm, Agilent Technologies). The mobile phases consisted of (A) 0.1% phosphoric acid in water and (B) 0.1% phosphoric acid in acetonitrile. The gradient elution program was as follows: 0–5 min, 90% A/10% B; 5–10 min, 87% A/13% B; 10–15 min, 86% A/14% B; 15–20 min, 78% A/22% B; 20–25 min, 78% A/22% B; 25–26 min, 0% A/100% B; 26–36 min, 0% A/100% B; 36–37 min, 90% A/10% B; 37–42 min, 90% A/10% B. The flow rate was 1 mL/min, column temperature was maintained at 35 °C, and detection was carried out at 275 nm.

#### 2.3.3. Isolation of Polymeric Tannin Fraction

Polymeric tannins were isolated using an HPLC system (Agilent 1100 Series, Agilent Technologies, Santa Clara, CA, USA) under conditions allowing early elution of monomers followed by selective collection of polymeric fractions. The mobile phases consisted of (A) 0.1% phosphoric acid in water/acetonitrile (65:35, *v*/*v*) and (B) 0.1% phosphoric acid in acetonitrile. The gradient program was as follows: 0–7 min, 100% A; 7–8 min, 0% A/100% B; 8–13 min, 0% A/100% B; 13–14 min, 100% A/0% B; and 14–18 min, 100% A. The flow rate was 1 mL/min, the column temperature was maintained at 35 °C, and the detection wavelength was 275 nm.

#### 2.3.4. Thioacidolysis and HPLC Analysis of Polymeric Tannins for mDP Determination

Purified polymeric tannin fractions were subjected to thioacidolysis using 0.2 M HCl in methanol containing 5% (*v*/*v*) toluene-α-thiol at 60 °C for 12 min. After reaction, samples were dried under nitrogen and re-dissolved for HPLC analysis. Thioacidolysis products were analyzed using the same column and mobile phases as described above, with an extended gradient program: 0–5 min, 90% A/10% B; 5–10 min, 87% A/13% B; 10–15 min, 86% A/14% B; 15–16 min, 78% A/22% B; 16–23 min, 78% A/22% B; 23–24 min, 75% A/25% B; 24–39 min, 40% A/60% B; 39–44 min, 30% A/70% B; 44–45 min, 0% A/100% B; 45–55 min, 0% A/100% B; 55–56 min, 90% A/10% B; 56–61 min, 90% A/10% B.

This approach enabled simultaneous detection of flavan-3-ol monomers and benzylthioether derivatives within a single run. The relative proportions of terminal and extension units were used to calculate the mean degree of polymerization (mDP). The mDP was calculated as

mDP = 1 + (Σ peak area of flavan-3-ol BT derivatives/Σ peak area of terminal flavan-3-ol monomers),

where the BT derivatives represent extension units released by thioacidolysis and the flavan-3-ol monomers represent terminal units. Because authentic standards for C-BT and EC-BT were not available, these benzylthioether derivatives were evaluated based on their chromatographic peak responses rather than by absolute quantification. For relative subunit analysis, the chromatographic contributions of C and EC within the terminal-unit fraction were calculated as C/(C + EC) and EC/(C + EC), respectively, whereas those of C-BT and EC-BT within the extension-unit fraction were calculated as C-BT/(C-BT + EC-BT) and EC-BT/(C-BT + EC-BT), respectively, for each biological replicate.

### 2.4. Statistical Analysis

All data are expressed as the mean ± standard deviation (SD) of three biological replicates. For comparisons among cultivars, two-way analysis of variance (ANOVA) was performed using the nine developmental time points common to all three cultivars (1 day and 1, 2, 4, 5, 6, 8, 10, and 13 weeks after flowering), with cultivar and developmental time as fixed factors, and their interaction was also evaluated. Developmental-stage differences within each cultivar were additionally evaluated by one-way ANOVA followed by Dunnett’s multiple comparison test, using the 1-day-after-flowering group as the reference control. Statistical significance was accepted at *p* < 0.05. Statistical analyses were performed using GraphPad Prism 7.

## 3. Results

### 3.1. Developmental Changes in Spectrophotometric Characteristics

**Figure 1:**
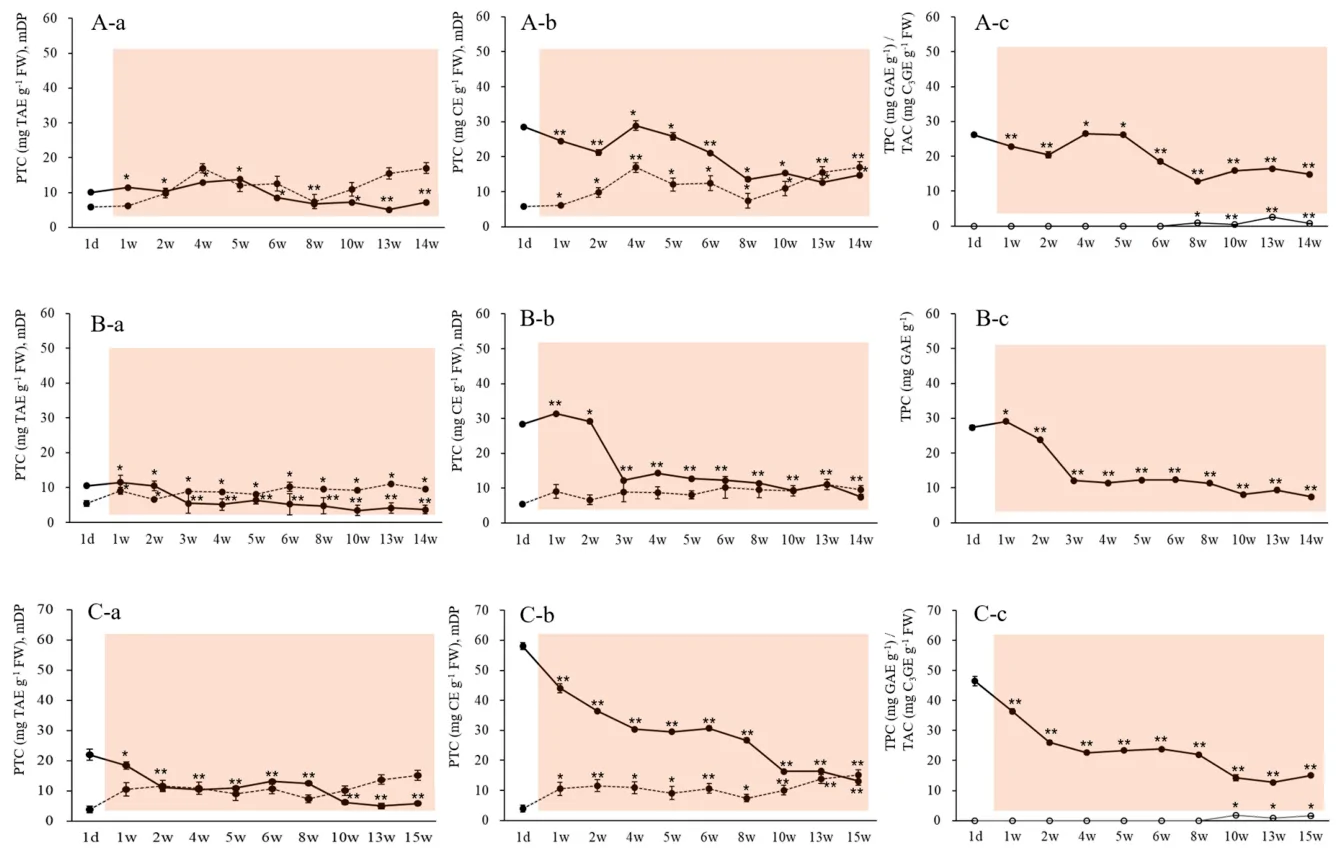
Developmental changes in phenolic compounds, anthocyanins, and mean degree of polymerization (mDP) in the berry skins of three table grape cultivars. (**A**) ‘Rosetta’, (**B**) ‘Shooting Star’, and (**C**) ‘Hongju Seedless’. (**A**-**a**), (**B**-**a**), and (**C**-**a**) Polymeric tannin content (PTC; solid line with closed circles) and mDP (dashed line with closed circles). (**A**-**b**), (**B**-**b**), and (**C**-**b**) Proanthocyanidin content (PAC; solid line with closed circles) and mDP (dashed line with closed circles). (**A**-**c**), (**B**-**c**), and (**C**-**c**) Total phenolic content (TPC; solid line with closed circles) and total anthocyanin content (TAC; solid line with open circles). PTC, PAC, TPC, and TAC are expressed as mg tannic acid equivalents (TAE), mg catechin equivalents (CE), mg gallic acid equivalents (GAE), and mg cyanidin-3-O-glucoside equivalents (C_3_GE) per g fresh weight, respectively. Data are presented as the mean ± SD of three independent biological replicates (*n* = 3). Asterisks indicate significant differences compared with the 1-day-after-flowering group according to Dunnett’s multiple comparison test (* *p* < 0.05, ** *p* < 0.01).

Developmental changes in total phenolic content (TPC), proanthocyanidin content (PAC), polymeric tannin content (PTC), and total anthocyanin content (TAC) in the skins of ‘Rosetta’ (RT), ‘Shooting Star’ (SS), and ‘Hongju Seedless’ (HS) are shown in Figure 1. Overall, all cultivars exhibited nonlinear changes in phenolic accumulation during berry development, although the magnitude and timing of these changes differed markedly among cultivars.

During the early developmental stage (1 day to 5 weeks after flowering), HS generally maintained higher levels of TPC, PAC, and PTC than RT and SS (Figure 1C). In RT, TPC and PAC were relatively high at the earliest stage, declined transiently, and then recovered to near-initial levels at 4–5 weeks, whereas PTC increased steadily and reached its maximum at 5 weeks (Figure 1A). In SS, TPC and PAC were also relatively high at the early stage but declined sharply by week 4 and remained at low levels thereafter; PTC followed a similar decreasing trend (Figure 1B).

During the mid-developmental stage (6–10 weeks after flowering), RT showed a pronounced decrease in TPC, PAC, and PTC after 5 weeks (Figure 1A). In contrast, SS maintained low and relatively stable levels of TPC, PAC, and PTC throughout the mid-developmental stage (Figure 1B). HS exhibited a more gradual decline than RT and SS and retained comparatively higher phenolic contents during the early-to-mid developmental period (Figure 1C).

Anthocyanin accumulation began after veraison and showed clear cultivar-dependent patterns. RT exhibited a marked increase in TAC and reached the highest level at 13 weeks, whereas HS showed moderate TAC accumulation beginning at 10 weeks (Figure 1A,C). In contrast, SS showed no detectable TAC throughout berry development (Figure 1B).

At the late developmental stage, from 13 weeks after flowering to harvest, differences among cultivars became more evident. In RT, TPC, PAC, and PTC, which had declined after the mid-developmental stage, increased again toward maturity (Figure 1A). HS also showed a slight recovery after reaching a minimum at 13 weeks, with modest increases in TPC, PAC, and PTC during the later stage (Figure 1C). In contrast, SS continued to decline after 13 weeks and showed the lowest levels of TPC, PAC, and PTC at the final developmental stage (Figure 1B).

Overall, HS was characterized by high early accumulation of phenolic and proanthocyanidin compounds, RT by intermediate accumulation followed by late-stage recovery and pronounced anthocyanin accumulation, and SS by a rapid decline and persistently low phenolic levels after the early developmental stage. Two-way ANOVA using the nine developmental time points common to all three cultivars revealed significant effects of cultivar, developmental time, and their interaction on TPC, PAC, PTC, and TAC (all *p* < 0.001; Table S2), demonstrating distinct cultivar-dependent developmental patterns in these phenolic parameters.

### 3.2. Flavan-3-Ol Composition Determined by HPLC

**Figure 2:**
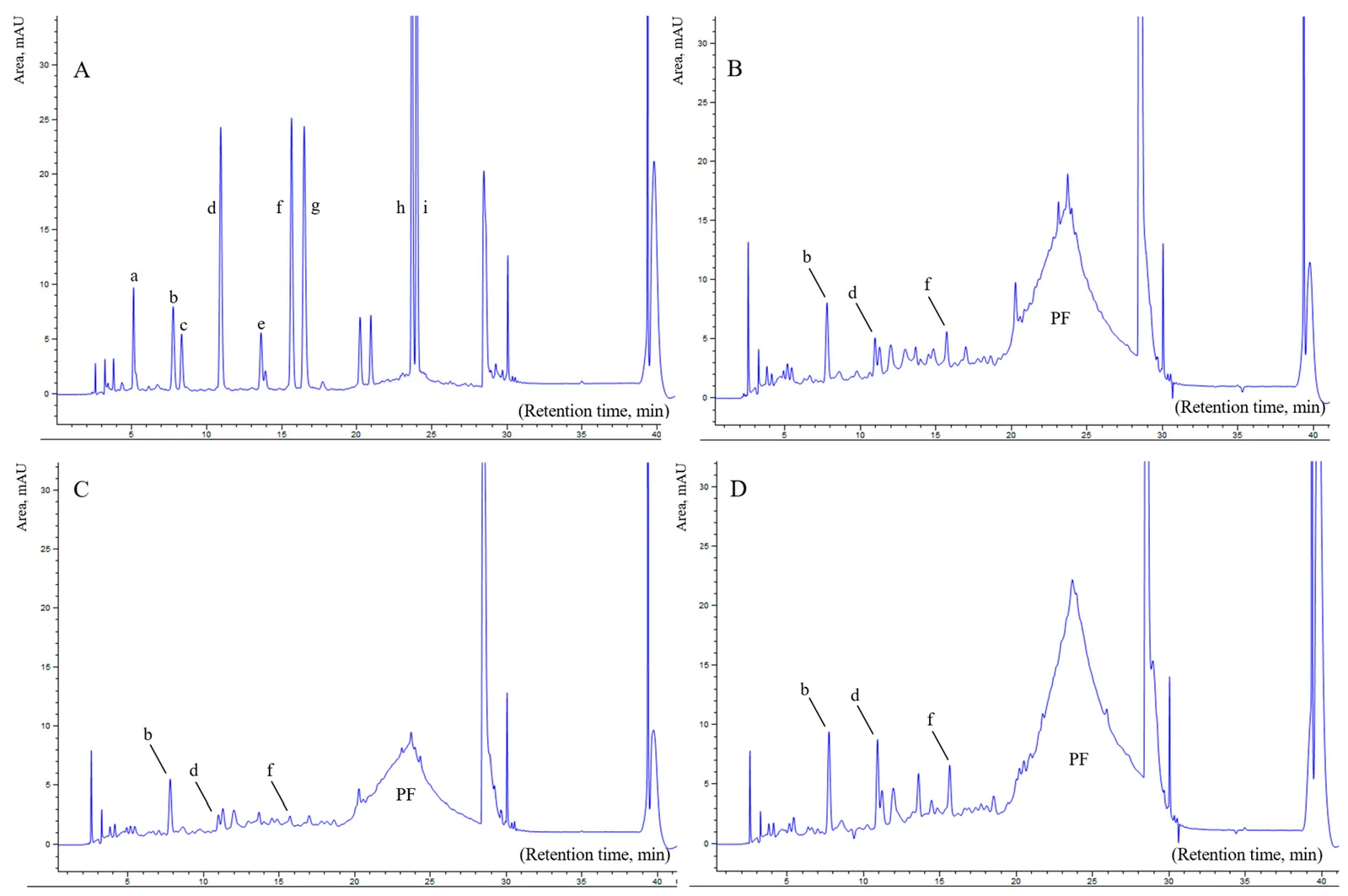
Representative HPLC chromatograms of flavan-3-ol standards and extracts from three table grape cultivars monitored at 275 nm. (**A**) Monomeric and dimeric flavan-3-ol standards (30 mg L^−1^). (**B**) ‘Rosetta’. (**C**) ‘Shooting Star’. (**D**) ‘Hongju Seedless’. Peaks: a, Gallocatechin (GC); b, Procyanidin B_1_ (B_1_); c, Epigallocatechin (EGC); d, Catechin (C); e, Procyanidin B_2_ (B_2_); f, Epicatechin (EC); g, Epigallocatechin gallate (EGCg); h, Epicatechin gallate (ECg); i, Catechin gallate (Cg); PF, Polymeric proanthocyanidin fraction.

Representative chromatograms of flavan-3-ol standards and grape skin extracts are presented in Figure 2. Under the applied chromatographic conditions, gallocatechin (GC) and epigallocatechin (EGC) eluted earlier than catechin (C) and epicatechin (EC). Among the non-galloylated monomers, C eluted before EC. Procyanidin B_1_ eluted before both C and EC, whereas the galloylated flavan-3-ols, epicatechin gallate (ECg) and catechin gallate (Cg), exhibited the longest retention times (Figure 2A).

Among the flavan-3-ol standards examined, only procyanidin B_1_, catechin, and epicatechin were detected in the grape skins of the three cultivars during berry development (Figure 2B–D). Galloylated flavan-3-ols, including ECg and Cg, were not detected at any developmental stage.

**Figure 3:**
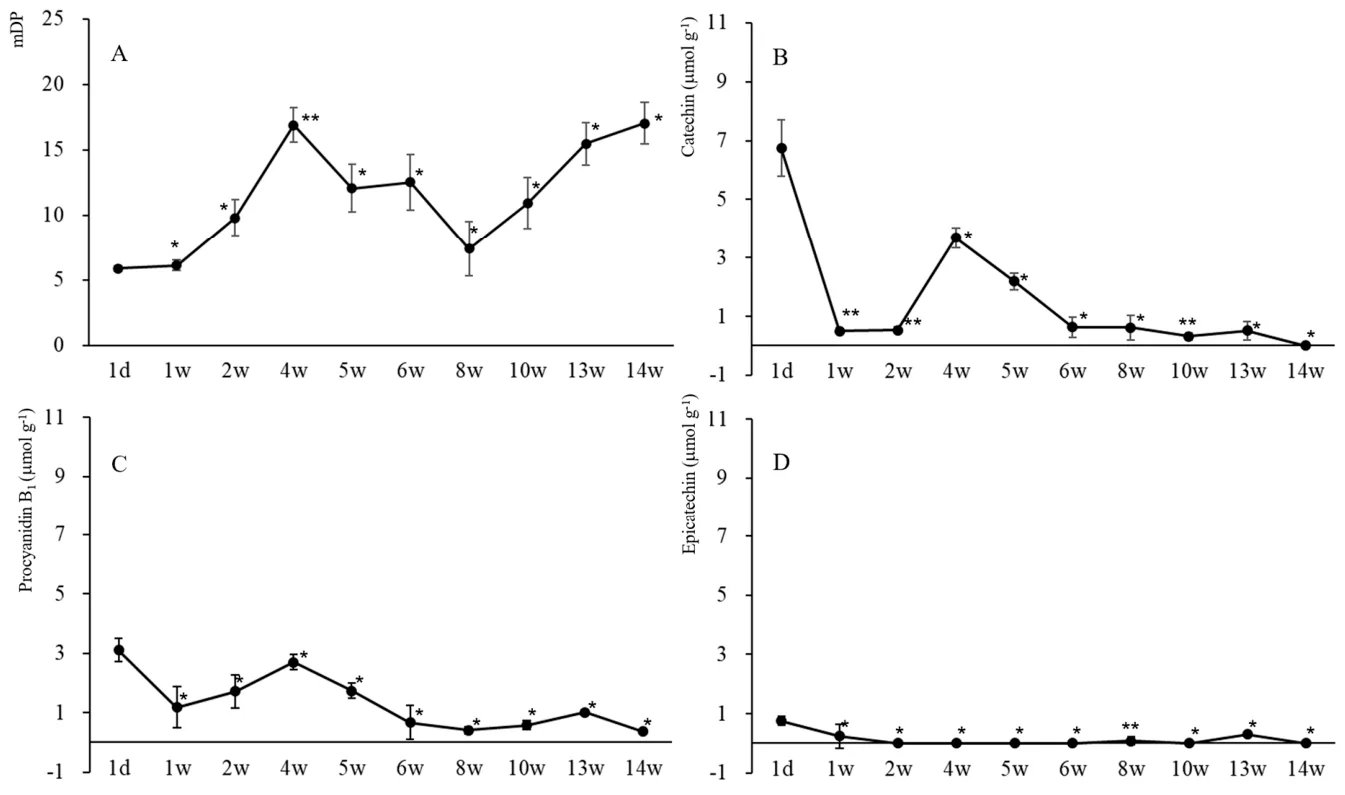
Developmental changes in mean degree of polymerization (mDP) and monomeric and dimeric flavan-3-ols in the berry skin of ‘Rosetta’ determined by HPLC-DAD. (**A**) Mean degree of polymerization (mDP). (**B**) Catechin. (**C**) Procyanidin B_1_. (**D**) Epicatechin. Flavan-3-ol concentrations are expressed as μmol g^−1^ fresh weight. Data are presented as the mean ± SD of three independent biological replicates (*n* = 3). Asterisks indicate significant differences compared with the 1-day-after-flowering group according to Dunnett’s multiple comparison test (* *p* < 0.05, ** *p* < 0.01).

Distinct cultivar-dependent temporal changes in flavan-3-ol composition were observed. In RT, procyanidin B_1_ was initially abundant, decreased after one week, transiently increased at four weeks, and then remained at relatively low levels until the late developmental stage before increasing again at maturity (Figure 3). Catechin showed a similar pattern, with high initial levels followed by a rapid decline, a transient increase at four weeks, and partial recovery at the final stage. Epicatechin was detected only at low levels during the earliest stage, disappeared during most of berry development, and reappeared at trace levels during ripening.

**Figure 4:**
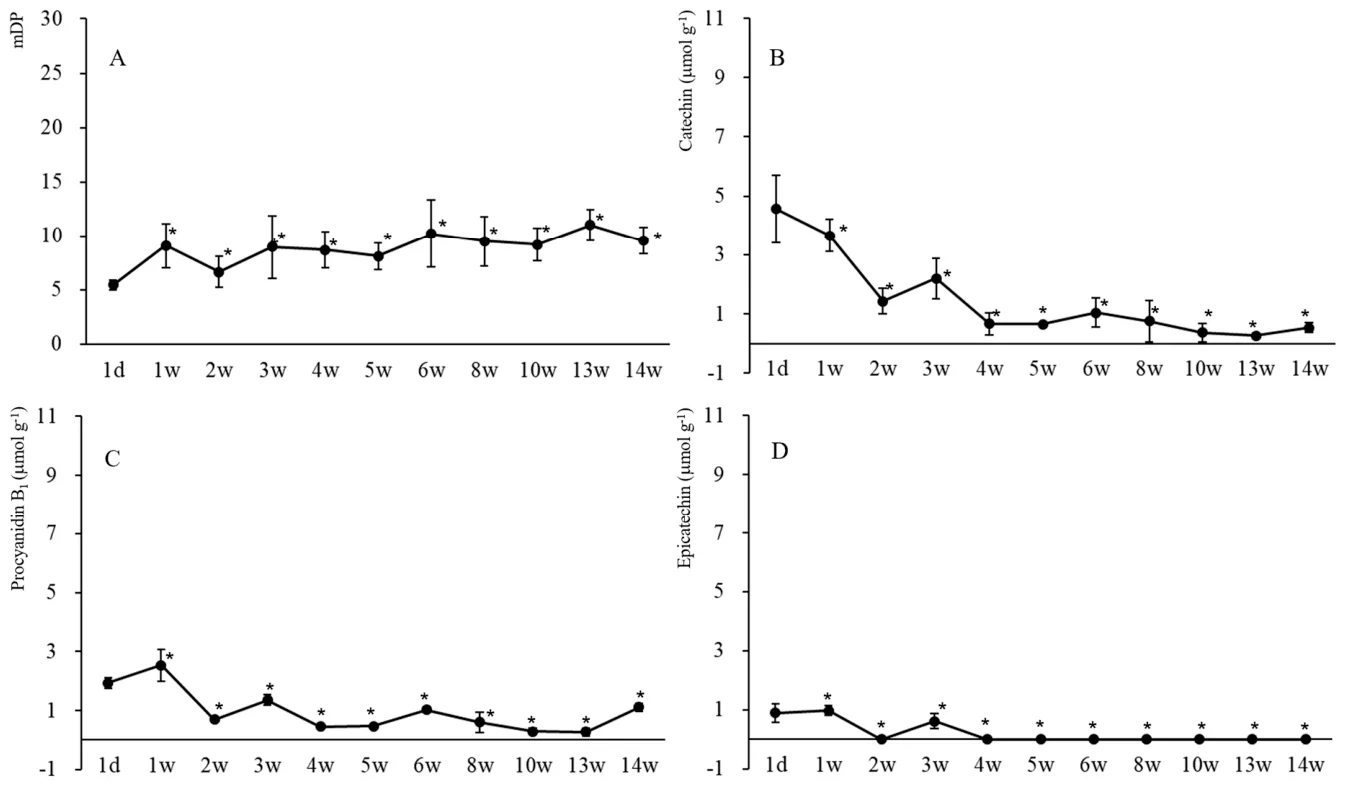
Developmental changes in mean degree of polymerization (mDP) and monomeric and dimeric flavan-3-ols in the berry skin of ‘Shooting Star’ determined by HPLC-DAD. (**A**) Mean degree of polymerization (mDP). (**B**) Catechin. (**C**) Procyanidin B_1_. (**D**) Epicatechin. Flavan-3-ol concentrations are expressed as μmol g^−1^ fresh weight. Data are presented as the mean ± SD of three independent biological replicates (*n* = 3). Asterisks indicate significant differences compared with the 1-day-after-flowering group according to Dunnett’s multiple comparison test (* *p* < 0.05).

SS exhibited a markedly different developmental pattern (Figure 4). Procyanidin B_1_ reached its maximum at one week but rapidly declined thereafter and remained at consistently low levels. Catechin also decreased sharply after the early stage, whereas epicatechin was detected only during the initial developmental period. Thus, extractable flavan-3-ol concentrations in SS remained comparatively low during the middle and late developmental stages.

**Figure 5:**
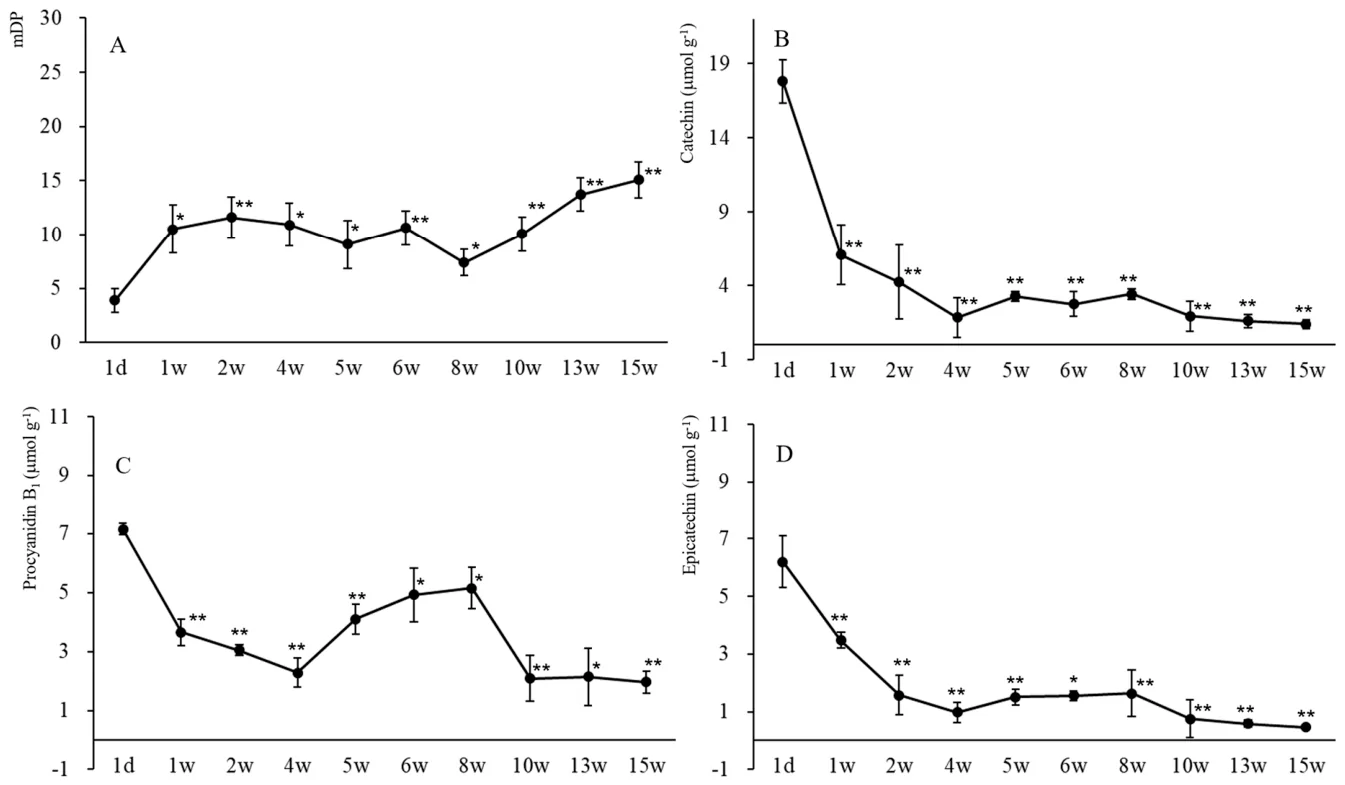
Developmental changes in mean degree of polymerization (mDP) and monomeric and dimeric flavan-3-ols in the berry skin of ‘Hongju Seedless’ determined by HPLC-DAD. (**A**) Mean degree of polymerization (mDP). (**B**) Catechin. (**C**) Procyanidin B_1_. (**D**) Epicatechin. Flavan-3-ol concentrations are expressed as μmol g^−1^ fresh weight. Data are presented as the mean ± SD of three independent biological replicates (*n* = 3). Asterisks indicate significant differences compared with the 1-day-after-flowering group according to Dunnett’s multiple comparison test (* *p* < 0.05, ** *p* < 0.01).

HS exhibited the highest concentrations of flavan-3-ol compounds during the early developmental stages among the three cultivars (Figure 5). Procyanidin B_1_, catechin, and epicatechin all showed high initial concentrations, followed by gradual decreases as berry development progressed. Although the overall trend was downward, the magnitude of decline was smaller than that observed in SS, and measurable concentrations were maintained during the middle developmental stages.

Comparison among cultivars therefore revealed clear differences in flavan-3-ol accumulation during berry development. HS exhibited the highest initial concentrations of catechin, epicatechin, and procyanidin B_1_; RT displayed intermediate concentrations together with transient fluctuations and partial recovery during ripening; and SS maintained comparatively low concentrations after the early developmental stages. Two-way ANOVA revealed significant effects of cultivar and developmental time on B_1_, C, and EC concentrations (all *p* < 0.001; Table S2). Significant cultivar × developmental time interactions were observed for B_1_ and EC (both *p* < 0.001), whereas the interaction was not significant for C (*p* = 0.159).

### 3.3. Structural Characterization of Polymeric Tannins by Fractionation and Thioacidolysis

**Figure 6:**
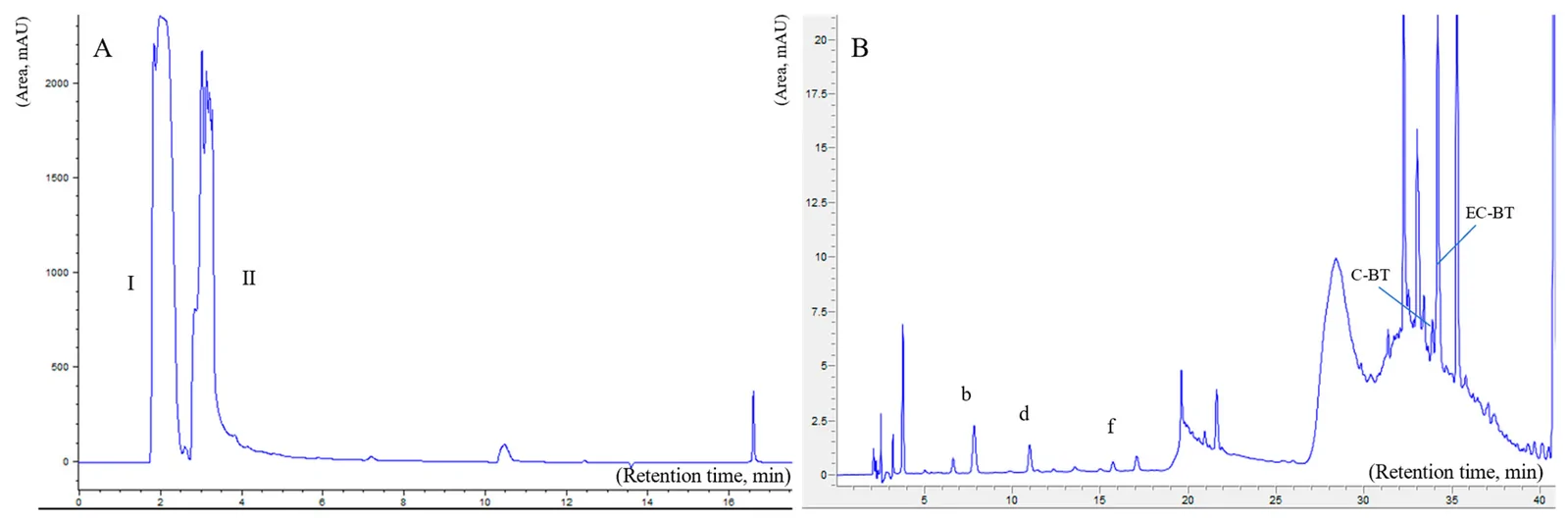
Representative HPLC chromatograms of (**A**) the polymeric proanthocyanidin fraction and (**B**) flavan-3-ols and their corresponding benzylthioether (BT) derivatives after thioacidolysis of the isolated polymeric tannin fraction from ‘Rosetta’. (I) Flavan-3-ol fraction, (II) polymeric proanthocyanidin fraction; b, procyanidin B1 (B1); d, catechin (C); f, epicatechin (EC); C-BT, catechin-benzylthioether; EC-BT, epicatechin-benzylthioether.

The chromatographic fractionation procedure was used to separate low-molecular-weight flavan-3-ols from the polymeric tannin fraction prior to thioacidolysis. Under the optimized gradient conditions, monomeric flavan-3-ols were eluted within approximately 3 min, whereas polymeric tannins were collected during the subsequent 3–8 min fraction (Figure 6A).

Representative chromatograms obtained after thioacidolysis are shown in Figure 6B. Acid-catalyzed cleavage in the presence of toluene-α-thiol produced two groups of compounds corresponding to flavan-3-ol terminal units and their benzylthioether (BT) derivatives originating from extension units. Catechin and epicatechin were detected at approximately 10.8 and 15.6 min, respectively, whereas their corresponding BT derivatives, C-BT and EC-BT, eluted at approximately 33.9 and 34.2 min, respectively. Procyanidin B_1_ was also detected in most samples following thioacidolysis. Because the samples subjected to thioacidolysis were obtained from the polymeric tannin fraction after prior elution of low-molecular-weight flavan-3-ols and dimers, the origin of the B_1_ detected after the reaction could not be unambiguously determined from the present chromatographic data.

Additional analysis of the relative chromatographic contributions of the detected subunits revealed variation in the relative subunit composition during berry development (Table S1). EC-BT was the predominant component of the extension-unit chromatographic response in RT and SS throughout development, whereas greater variability in the relative contributions of C-BT and EC-BT was observed in HS. The relative contributions of C and EC within the terminal-unit fraction also varied among developmental stages; however, no consistent developmental shift in terminal-unit composition was evident across the three cultivars.

Mean degree of polymerization (mDP) showed an overall tendency to increase as berry development progressed, although the magnitude and temporal patterns of the changes varied among cultivars. RT showed an apparent increase in mDP during the late developmental stages. In contrast, SS maintained relatively low and stable mDP values throughout development, whereas HS exhibited an intermediate pattern with a tendency toward a gradual increase accompanied by temporal fluctuations. However, two-way ANOVA using the nine developmental time points common to all three cultivars detected no significant effects of cultivar (*p* = 0.298), developmental time (*p* = 0.244), or their interaction (*p* = 0.453) on mDP (Table S2).

The observed developmental patterns of mDP did not completely parallel those of phenolic concentration or extractable flavan-3-ol abundance. RT combined partial recovery of extractable phenolics with a tendency toward higher mDP during ripening. HS maintained relatively high flavan-3-ol concentrations during early development while showing temporal changes in mDP, whereas SS exhibited both low flavan-3-ol concentrations and relatively limited variation in mDP.

## 4. Discussion

### 4.1. Cultivar-Dependent Developmental Changes in Grape Skin Phenolics

The results demonstrate that developmental changes in grape skin phenolics were strongly cultivar-dependent. This interpretation was supported by the two-way ANOVA, which revealed significant effects of cultivar, developmental time, and their interaction on TPC, PAC, PTC, and TAC (all *p* < 0.001; Table S2). HS was characterized by high early accumulation of phenolic and proanthocyanidin compounds, RT showed a more dynamic developmental pattern with late-stage recovery, and SS exhibited a rapid decline followed by persistently low phenolic levels. These differences are consistent with previous studies demonstrating that flavan-3-ol accumulation, polymerization, and changes in phenolic concentration during grape berry development are strongly influenced by genotype and developmental stage [13,15,24,28].

The high initial levels of TPC, PAC, and PTC in HS, together with its high concentrations of flavan-3-ols, indicate pronounced early accumulation of flavan-3-ol-related phenolics compared with RT and SS. Similar genotype-dependent differences in phenolic accumulation have previously been reported among grape cultivars [28,29]. Conversely, the rapid decline in TPC, PAC, and PTC in SS indicates limited retention of extractable phenolic compounds after the early developmental stage.

The decrease in TPC, PAC, and PTC observed during the middle developmental stage, particularly in RT, may be partly associated with dilution caused by berry enlargement. Changes in tannin extractability may also contribute to this pattern because progressive interactions between tannins and cell wall polysaccharides can reduce tannin extractability during berry development [30,31]. Similar immobilization of tannins through interactions with cell wall components has been reported in grape and other fruits [31,32]. Thus, decreases in measured phenolic concentration during berry development should not necessarily be interpreted solely as decreases in tannin abundance.

Anthocyanin accumulation provided an additional distinction among the three cultivars. RT showed pronounced TAC accumulation after veraison, HS showed a more moderate increase, whereas no detectable TAC was observed in SS. Anthocyanins and flavan-3-ols share common precursors within the phenylpropanoid and flavonoid biosynthetic pathways, and developmental changes in flavonoid metabolism after veraison may therefore influence the relative accumulation of tannin-related compounds and anthocyanin pigments [22,33]. The concurrent decrease in TPC, PAC, and PTC and increase in TAC observed in RT and, to a lesser extent, HS are consistent with such developmental changes in flavonoid metabolism. However, because metabolic flux and gene expression were not directly examined in the present study, these changes cannot establish direct metabolic redirection from flavan-3-ols toward anthocyanin biosynthesis.

The late-stage recovery of TPC, PAC, and PTC in RT and, to a lesser extent, HS further demonstrates that changes in extractable phenolics during ripening are not necessarily unidirectional. Changes in extractability and structural characteristics of grape skin tannins during ripening have previously been reported [14]. Collectively, these results indicate that developmental patterns of grape skin phenolics reflect the interaction between developmental stage and cultivar-specific characteristics.

### 4.2. Cultivar-Dependent Changes in Flavan-3-Ol Composition

The chromatographic behavior of the flavan-3-ol standards was consistent with their molecular characteristics. GC and EGC eluted earlier than C and EC, reflecting the greater polarity associated with additional hydroxyl groups on the B-ring under reversed-phase chromatographic conditions [34,35]. In contrast, the galloylated flavan-3-ols ECg and Cg showed longer retention, consistent with increased hydrophobic interactions of the galloyl moiety with the stationary phase [36].

Only procyanidin B_1_, catechin, and epicatechin were detected as extractable flavan-3-ols in the skins of the three cultivars, whereas ECg and Cg were not detected. Galloylated flavan-3-ol units have been reported as structural components of proanthocyanidins in several wine grape cultivars [12,37]. Therefore, the predominance of non-galloylated extractable flavan-3-ols observed in the present study may reflect cultivar- or genotype-dependent differences in flavan-3-ol composition. Importantly, however, the absence of ECg and Cg as detectable free monomers does not necessarily indicate their complete absence from polymeric tannin structures.

The developmental patterns of individual flavan-3-ols broadly corresponded with those observed spectrophotometrically. HS exhibited the highest initial concentrations of procyanidin B_1_, catechin, and epicatechin and retained measurable levels during subsequent development. In contrast, SS showed a rapid decrease after the early developmental stage, whereas RT displayed more pronounced temporal fluctuations and partial recovery toward maturity.

Two-way ANOVA further revealed significant effects of cultivar and developmental time on B_1_, C, and EC concentrations (all *p* < 0.001; Table S2). Significant cultivar × developmental time interactions were observed for B_1_ and EC (both *p* < 0.001), whereas the interaction was not significant for C (*p* = 0.159). Similar genotype-dependent differences in flavan-3-ol accumulation have been reported among grape cultivars with contrasting phenolic characteristics [28,29,38,39]. Flavan-3-ol monomers occur both as free phenolic compounds and as structural building blocks of condensed tannins, and their relative abundance may change during berry development in association with tannin formation and other developmental processes [12,13,40].

Consequently, the decline in extractable monomeric flavan-3-ols observed during development may reflect several processes, including their incorporation into polymeric structures and decreased extractability associated with interactions with cell wall polysaccharides [30,31,41]. The cultivar-dependent variation observed in the present study therefore reflects differences in the abundance of extractable flavan-3-ols and may also be associated with differences in their contribution to polymeric tannin development.

### 4.3. Developmental Changes in Polymeric Tannin Structure

Selective isolation of the polymeric tannin fraction before thioacidolysis was important for minimizing interference from low-molecular-weight flavan-3-ols, which can affect determination of terminal and extension units and consequently mDP estimation [37,42,43,44]. The chromatographic separation of monomeric flavan-3-ols from the subsequently collected polymeric fraction (Figure 6A) minimized contamination by low-molecular-weight compounds, consistent with previous approaches used for tannin depolymerization analysis [45].

Following thioacidolysis, catechin and epicatechin were detected as terminal units, whereas C-BT and EC-BT represented extension units (Figure 6B). The chromatographic profiles of these products were consistent with those previously reported for depolymerized grape tannins [37,43,45,46]. However, procyanidin B_1_ was also detected in most samples after thioacidolysis. Because low-molecular-weight flavan-3-ols and dimers had been eluted before collection of the polymeric tannin fraction, the detected B_1_ could not be readily attributed to the presence of initially extractable B_1_ in the fraction subjected to thioacidolysis. Nevertheless, its origin could not be unambiguously established from the present data. If the detected B_1_ represented incomplete cleavage of oligomeric or polymeric material, the corresponding terminal- and extension-unit responses would not be fully represented in the peak areas used for mDP calculation, thereby potentially introducing bias into the estimated mDP. The magnitude and direction of this potential bias could not be determined from the present data and should therefore be considered a limitation of the mDP estimates.

Analysis of the relative chromatographic contributions of the thioacidolysis products provided additional information on the subunit composition of the polymeric tannins. EC-BT predominated in the extension-unit chromatographic response of RT and SS throughout development, whereas HS showed greater variation in the relative contributions of C-BT and EC-BT. In contrast, the relative contributions of the terminal units C and EC varied among developmental stages without a consistent pattern across cultivars. These observations indicate that developmental changes in condensed tannin structure cannot be described solely by changes in mDP, because tannins with similar average degrees of polymerization may differ in their relative terminal- and extension-unit composition. However, because authentic standards for C-BT and EC-BT were unavailable, these relative chromatographic contributions should be interpreted as compositional patterns based on peak responses rather than as absolute or molar subunit proportions.

The observed mDP values showed an overall tendency to increase during berry development, suggesting possible progressive changes in condensed tannin polymer size as the berries matured. This tendency is consistent with previous reports of increasing tannin polymerization during grape berry development [12,16,47]. However, two-way ANOVA detected no significant effects of cultivar (*p* = 0.298), developmental time (*p* = 0.244), or their interaction (*p* = 0.453) on mDP (Table S2). Therefore, the cultivar-specific patterns observed in the present study should be interpreted as descriptive trends. RT showed an apparent increase in mDP during late development, HS showed a tendency toward a gradual increase with temporal fluctuations, whereas SS maintained relatively low and stable mDP values.

Importantly, the observed patterns in mDP did not simply parallel those in total phenolic concentration or extractable flavan-3-ol abundance. These measurements represent different, although structurally related, aspects of condensed tannin development. Extractable monomeric flavan-3-ols represent free low-molecular-weight compounds that can also serve as structural building blocks of condensed tannins, whereas the polymeric tannin fraction represents the higher-molecular-weight tannin material recovered from the skin. Within these polymers, the relative contributions of terminal and extension subunits provide information on subunit composition, while mDP reflects the average relationship between extension and terminal units and therefore provides an estimate of average polymer size.

Consequently, a decrease in extractable monomer concentration does not necessarily result in a proportional increase in the recovered polymeric tannin fraction or mDP, and changes in mDP can occur without corresponding changes in total extractable tannin concentration. RT showed an apparent increase in mDP during late development despite substantial earlier decreases in extractable phenolics. HS combined high early flavan-3-ol concentrations with a tendency toward a gradual increase in mDP, whereas SS exhibited both low flavan-3-ol concentrations and relatively limited variation in mDP. These observations suggest that tannin abundance, subunit composition, and average polymer size represent related but distinct characteristics of condensed tannin development.

Changes in tannin extractability may further contribute to the apparent relationship between tannin abundance and polymer size. Interactions between tannins and cell wall polysaccharides can reduce extractability during grape berry maturation [31,32]. Therefore, changes in measured tannin concentration cannot necessarily be interpreted as equivalent to changes in tannin polymerization.

Taken together, the spectrophotometric, HPLC, and thioacidolysis results indicate that phenolic accumulation, flavan-3-ol composition, polymeric tannin abundance, and mDP exhibited related but not identical patterns during berry development. Cultivar-dependent developmental patterns were evident for phenolic and flavan-3-ol parameters, whereas two-way ANOVA detected no significant effects of cultivar, developmental time, or their interaction on mDP. RT showed an apparent increase in mDP during late development, HS showed a tendency toward a gradual increase with temporal fluctuations, and SS maintained relatively low and stable mDP values. Thus, comprehensive characterization of condensed tannin development in table grape skins requires integration of quantitative phenolic measurements with compositional and structural analyses.

## 5. Conclusions

This study characterized developmental patterns in phenolic compounds and structural characteristics of condensed tannins in the berry skins of three table grape cultivars, ‘Rosetta’, ‘Shooting Star’, and ‘Hongju Seedless’, from flowering to harvest. Total phenolics, proanthocyanidins, polymeric tannins, and flavan-3-ol composition exhibited distinct cultivar-dependent developmental patterns, whereas mDP showed descriptive temporal trends without significant effects of cultivar, developmental time, or their interaction.

‘Hongju Seedless’ showed high early flavan-3-ol concentrations with measurable levels retained during subsequent development and temporal variation in mDP, whereas ‘Rosetta’ exhibited an apparent increase in mDP during maturation. In contrast, ‘Shooting Star’ exhibited comparatively low tannin accumulation and relatively limited variation in mDP. These results indicate that condensed tannin development in table grape skins involves both quantitative and structural characteristics that do not necessarily change in parallel during berry development.

The present findings provide fundamental information on phenolic and tannin development in newly developed Korean table grape cultivars and may serve as a basis for future studies investigating the physiological and quality-related roles of condensed tannins during berry development and ripening.

## Data Availability

The original contributions presented in this study are included in the article/Supplementary Materials. Further inquiries can be directed to the corresponding author.

## Supplementary Materials

The following supporting information can be downloaded at https://www.mdpi.com/article/10.3390/foods15193482/s1. Table S1. Relative chromatographic contributions of terminal and extension subunits during berry skin development in three table grape cultivars. Table S2. Two-way ANOVA for the effects of cultivar, developmental time, and their interaction on phenolic parameters, flavan-3-ol concentrations, and mean degree of polymerization (mDP) in grape skins.
