# Supplementary material for: Developmental Changes in Phenolic Composition and Condensed Tannin Structure in the Skins of Three Table Grape Cultivars: ‘Rosetta’, ‘Shooting Star’, and ‘Hongju Seedless’
Source: Foods. 2026 Sep 29;15(19):3482. doi: 10.3390/foods15193482 (PMC13664545; doi:10.3390/foods15193482)
Supplement: Supplementary file 1 [file foods-15-03482-s001.zip › foods-4563302-supplementary.pdf]

Table S1. Relative chromatographic contributions of terminal and extension subunits during berry skin development in three table grape cultivars.

Cultivar	Developmental stage	Terminal C (%)	Terminal EC (%)	Extension C-BT (%)	Extension EC-BT (%)
RT	1 day after flowering	50.88 ± 1.11	49.12 ± 1.11	6.64 ± 0.07	93.36 ± 0.07
RT	1 week after flowering	53.44 ± 1.16	46.56 ± 1.16	6.77 ± 0.31	93.23 ± 0.31
RT	2 weeks after flowering	51.84 ± 2.19	48.16 ± 2.19	6.98 ± 0.39	93.02 ± 0.39
RT	4 weeks after flowering	61.71 ± 28.42	38.29 ± 28.42	5.47 ± 1.95	94.53 ± 1.95
RT	5 weeks after flowering	51.56 ± 1.83	48.44 ± 1.83	6.55 ± 0.67	93.45 ± 0.67
RT	6 weeks after flowering	65.53 ± 10.22	34.47 ± 10.22	5.04 ± 1.57	94.96 ± 1.57
RT	8 weeks after flowering	58.45 ± 15.87 (n = 2)	41.55 ± 15.87 (n = 2)	7.36 ± 1.50	92.64 ± 1.50
RT	10 weeks after flowering	n.d.	n.d.	6.92 ± 1.63	93.08 ± 1.63
RT	13 weeks after flowering	62.43 ± 10.77	37.57 ± 10.77	6.73 ± 2.97	93.27 ± 2.97
RT	14 weeks after flowering	56.70 ± 10.57	43.30 ± 10.57	9.75 ± 3.53	90.25 ± 3.53
SS	1 day after flowering	58.87 ± 10.05	41.13 ± 10.05	6.94 ± 2.98	93.06 ± 2.98
SS	1 week after flowering	58.57 ± 11.09	41.43 ± 11.09	6.91 ± 2.46	93.09 ± 2.46
SS	2 weeks after flowering	68.22 ± 4.76	31.78 ± 4.76	6.60 ± 1.77	93.40 ± 1.77
SS	3 weeks after flowering	81.82 ± 25.71 (n = 2)	18.18 ± 25.71 (n = 2)	5.22 ± 1.82	94.78 ± 1.82
SS	4 weeks after flowering	55.38 ± 5.85	44.62 ± 5.85	7.75 ± 1.24	92.25 ± 1.24
SS	5 weeks after flowering	75.72 ± 21.04	24.28 ± 21.04	6.66 ± 0.91	93.34 ± 0.91
SS	6 weeks after flowering	66.29 ± 11.33	33.71 ± 11.33	5.25 ± 1.03	94.75 ± 1.03
SS	8 weeks after flowering	67.50 ± 13.42	32.50 ± 13.42	5.78 ± 0.47	94.22 ± 0.47
SS	10 weeks after flowering	59.42 ± 15.10	40.58 ± 15.10	6.26 ± 1.41	93.74 ± 1.41
SS	13 weeks after flowering	60.87 ± 3.46	39.13 ± 3.46	6.41 ± 1.98	93.59 ± 1.98
SS	14 weeks after flowering	56.79 ± 4.57	43.21 ± 4.57	5.70 ± 0.36	94.30 ± 0.36
HS	1 day after flowering	49.14 ± 4.09	50.86 ± 4.09	26.76 ± 5.02	73.24 ± 5.02
HS	1 week after flowering	56.47 ± 13.44	43.53 ± 13.44	66.25 ± 29.02	33.75 ± 29.02
HS	2 weeks after flowering	57.41 ± 5.04	42.59 ± 5.04	72.74 ± 19.60	27.26 ± 19.60
HS	4 weeks after flowering	52.57 ± 11.48	47.43 ± 11.48	45.32 ± 27.20	54.68 ± 27.20
HS	5 weeks after flowering	48.95 ± 6.81	51.05 ± 6.81	27.22 ± 10.86	72.78 ± 10.86
HS	6 weeks after flowering	53.05 ± 4.31 (n = 2)	46.95 ± 4.31 (n = 2)	13.56 ± 13.79	86.44 ± 13.79

HS	8 weeks after flowering	52.91 ± 7.57	47.09 ± 7.57	44.71 ± 18.38	55.29 ± 18.38
HS	10 weeks after flowering	64.59 ± 1.69 (n = 2)	35.41 ± 1.69 (n = 2)	41.57 ± 36.01	58.43 ± 36.01
HS	13 weeks after flowering	52.82 ± 7.20	47.18 ± 7.20	34.19 ± 14.59	65.81 ± 14.59
HS	15 weeks after flowering	52.72 ± 5.61	47.28 ± 5.61	35.32 ± 22.63	64.68 ± 22.63

Note: Values are expressed as mean ± SD. Unless otherwise indicated, $n = 3$ biological replicates. Relative chromatographic contributions of terminal units were calculated as C/(C + EC) × 100 and EC/(C + EC) × 100, and those of extension units as C-BT/(C-BT + EC-BT) × 100 and EC-BT/(C-BT + EC-BT) × 100. RT, 'Rosetta'; SS, 'Shooting Star'; HS, 'Hongju Seedless'**;** C, catechin; EC, epicatechin; C-BT, catechin-benzylthioether; EC-BT, epicatechin-benzylthioether; n.d., not determined because terminal-unit peak-area contributions were unavailable. Because authentic standards for C-BT and EC-BT were unavailable, the values represent relative chromatographic peak-area contributions and should not be interpreted as absolute concentrations or molar subunit proportions.

Table S2. Two-way ANOVA for the effects of cultivar, developmental time, and their interaction on phenolic parameters, flavan-3-ol concentrations, and mean degree of polymerization (mDP) in grape skins.

**Parameter**	**Cultivar F**	***p***	**Developmental time F**	***p***	**Cultivar × developmental time F**	***p***
TPC	1724.37	< 0.001	1380.26	< 0.001	235.31	< 0.001
PAC	3550.97	< 0.001	1570.13	< 0.001	251.18	< 0.001
PTC	232.80	< 0.001	112.54	< 0.001	28.59	< 0.001
TAC	7872.70	< 0.001	9254.47	< 0.001	5265.04	< 0.001
B1	85.74	< 0.001	9.98	< 0.001	4.61	< 0.001
C	11.18	< 0.001	11.36	< 0.001	1.44	0.159
EC	96.40	< 0.001	16.44	< 0.001	5.00	< 0.001
mDP	1.24	0.298	1.34	0.244	1.02	0.453

Note: Two-way ANOVA was performed using the nine developmental time points common to all three cultivars (1 day and 1, 2, 4, 5, 6, 8, 10, and 13 weeks after flowering), with cultivar and developmental time as fixed factors, including their interaction. Degrees of freedom were 2 for cultivar, 8 for developmental time, 16 for the cultivar × developmental time interaction, and 54 for residual error. TPC, total phenolic content; PAC, proanthocyanidin content; PTC, polymeric tannin content; TAC, total anthocyanin content; B_1, procyanidin B_1; C, catechin; EC, epicatechin; mDP, mean degree of polymerization.
